# Occurrence of natural enemies of fall armyworm, *Spodoptera frugiperda* (Lepidoptera: Noctuidae) in Nigeria

**DOI:** 10.1371/journal.pone.0254328

**Published:** 2021-07-12

**Authors:** Akindele Oluwole Ogunfunmilayo, Shakiru Adewale Kazeem, Joy Ejemen Idoko, Raphael Abiodun Adebayo, Elizabeth Yetunde Fayemi, Okuyinka Bosola Adedibu, Qudrah Olaitan Oloyede-Kamiyo, Joy Oluchi Nwogwugwu, Oluwafolake Adenike Akinbode, Shina Salihu, Lisa Clare Offord, Alan Graham Buddie, Thomas Inomisan Ofuya

**Affiliations:** 1 Post-Entry Quarantine, Surveillance and Diagnostic Station, Nigeria Agricultural Quarantine Service, Ibadan, Oyo State, Nigeria; 2 Department of Crop, Soil and Pest Management, Federal University of Technology, Akure, Ondo State, Nigeria; 3 Maize Improvement Programme for Southern Ecology of Institute of Agricultural Research and Training, Obafemi Awolowo University, Ibadan, Oyo State, Nigeria; 4 Forest Research Institute of Nigeria, Ibadan, Nigeria; 5 National Cereal Research Institute, Ibadan, Nigeria; 6 CABI, Egham, United Kingdom; Northwest A&F University, CHINA

## Abstract

Fall armyworm (FAW; *Spodoptera frugiperda*), an exotic moth which recently invaded Africa, is a highly destructive pest of cereals especially maize a highly valued staple crop in Nigeria. The use of natural enemies such as predators or parasitoids for FAW control is more economically viable and environmentally safer than currently recommended synthetic insecticides. Natural enemies to combat the pest have not yet been reported in Nigeria. An exploration for the pests’ natural enemies was undertaken by collecting FAW eggs and larvae from maize fields. These were reared in the laboratory for emergence, identification and efficacy as natural enemies. This yielded *Euplectrus laphygmae* (Hymenoptera: Eulophidae); *Telenomus remus* (Hymenoptera: Platygastridae) and *Trombidium* sp. (Acari.: Trombidiidae). *Cotesia* or *Apanteles* spp. were inferred to occur since *Stictopisthus* sp. (Hym.: Ichneumonidae), a secondary parasitoid, that attacks cocoons of Microgasterinae (e.g. *Cotesia*, *Apanteles* etc.) also emerged. Species of yet-to-be identified predators were also observed in various niches of maize plants. A positive relationship was found between FAW instar and the number of *E*. *laphygmae* eggs/instar ranging, on average, from 1.5 on second instar to 5.5 on fourth instars hosts. Parasitism rate of *T*. remus on FAW eggs was 100%. Parasitic mite infestation resulted in increasing paleness, reduced feeding, growth and movement as well as death of FAW 1^st^ instars. Thus, the occurrence of FAW natural enemies in Nigeria calls for advocacy campaign to incorporate their use into integrated pest management strategies that attract and allow natural enemies to thrive for FAW management.

## 1. Introduction

The fall armyworm (FAW) (*Spodoptera frugiperda* JE Smith (Lepidoptera: Noctuidae) is a pest of more than 80 different crops that was previously confined to native regions in North and South America and was recorded in Nigeria for the first time in 2016 [1]. It has also been reported in several other sub-Saharan African countries [2, 3]. In the absence of proper control measures of FAW, maize yield loss is estimated to be about US$13 billion per annum throughout sub-Saharan Africa [4].

Since its first detection in Nigeria in 2016 [1], several efforts have been devoted to creating awareness of the pest problem and implementing control measures, with emphasis on the use of synthetic insecticides hitherto not used in maize cultivation. These efforts have lessened the yield impact of FAW compared to the level of damage during the early period of its introduction into Nigeria (Ogunfunmilayo *et al*. unpublished data). Unlike the intensively farmed commercial scale fields, maize farms owned by subsistence farmers have little or no input of synthetic insecticide due to affordability. Hence, we hypothesize that some other agents, such as natural enemies, may be contributing to the reduced FAW damage observed in these subsistence farms.

The use of natural enemies for biological control of insect pests has not been fully explored. Natural enemies of key agricultural pests offer an economically sustainable and environmentally safer alternative to synthetic insecticides, if they can be identified and incorporated into an integrated pest management (IPM) system. A wide array of natural enemies such as entomopathogens, parasitoids and predators have been reported for biological control of FAW in its native region and several other host countries [2–11]. Augmentative release of native species or introduction of imported exotic species of parasitoids and predators are environmentally safe management options for introduced exogenous insect pests. Parasitoids spend at least one stage of their life in an intimate association with specific life stages of the host pest. They attack either the eggs (egg parasitoids) or larvae (larval parasitoids) of the host. The development of larvae stage of the parasitoids results in death of the insect host [2, 4]. Predators can kill all insect life stages but they do not live on the host [2].

Different species of parasitoid and predator are known to attack FAW [2, 5, 9] in the Americas and the Caribbean islands [5]. In Africa, parasitoids such as *Cotesia icipe* Fernandez-Triana & Fiobe (Hymenoptera: Braconidae), *Palexorista zonata* (Curran) (Diptera: Tachinidae), *Charops ater* Szepligeti (Hymenoptera: Ichneumonidae), *Chelonus curvimaculatus* Cameron (Hymenoptera: Braconidae) and *Coccygidium luteum* (Brulle) (Hymenoptera: Braconidae), *Trichogramma* spp. (Hymenoptera: Trichogrammatidae) and *Telenomus* spp. attack FAW [2–4, 11–13]. Prasanna *et al*. [2] also listed *Coleomegilla maculata* De Geer (Coleoptera: Coccinellidae); *Hippodamia convergens* Guerin-Meneville (Coleoptera: Coccinellidae); *Cycloneda sanguinea* Linneaus (Coleoptera: Coccinellidae); *Zelus* spp. Fabricius (Hymenoptera: Reduviidae); *Euborellia annulipes* Lucas (Dermaptera: Anisolabididae); *Doru luteipes* Scudder (Dermaptera: Forficulidae); *Podisus maculiventris* Say (Hemiptera: Pentatomidae); *Calosoma granulatum* Perty (Coleoptera: Carabidae); *Geocoris punctipes* Say (Hemiptera: Geocoridae) and *Orius insidiosus* Say (Hemiptera: Anthocoridae) as predators of FAW.

Since FAW is a relatively new insect pest in Nigeria, there is a dearth of information on its natural enemies in the country. The goal of this study is to determine the occurrence of FAW natural enemies in Nigerian maize fields and assess their effectiveness under laboratory conditions for the purpose of designing biological control and IPM options.

## 2. Materials and methods

### 2.1. Insect collection and rearing

Maize fields intercropped with one or more combinations of cassava (*Manihot esculenta*), cocoyam (*Colocasia esculentus*), African spinach (*Celosia argentea*) and jute mallow (*Corchorus olitorius*) were visited at three-day intervals in different randomly selected locations in Ibadan, Oyo State, Nigeria (Table 1). An average of three farms per location were visited during the late rainy season after the initial detection of FAW parasitoids in the Gada location (Table 1). Fall armyworm (FAW) larvae and egg masses were sampled from 7 to 21 days old maize plants, with or without FAW damage signs, at 2–6 leaves (early whorl) stage. On each visit to the farms, less than 3 egg masses of approximately 75–185 eggs were observed and collected by cutting the part of the leaf on which the respective egg mass was laid, without dislodging the eggs. The eggs in the mass were counted using 10x electrical magnifying lens. Also an average of 100–150 larvae at different instar stages were randomly removed from the maize plants at the respective farms during each visit.

**Table 1:** Locations of maize farms visited.

Locations	Coordinates	Collection staring date	Host Plants
Gada	7.3839°N	15^th^- 28th October, 2019	Maize
	3.84556°E		
NCRI field 1	7.38674°N	2^nd^ - 23^rd^ Nov., 2019	Maize
	3.84514°E		
NAQS hostel	7.38369°N	1^st^-19 Nov., 2019	Maize
	3.83895°E		
IART	7.38652°N	29^th^ Oct.– 20^th^ Nov., 2019	Maize
	3.84608°E		
Omi-Adio	7.3900° N	29^th^ Oct. - 21 Nov., 2019	Maize
	3.7537°E		

**Fig 1:**
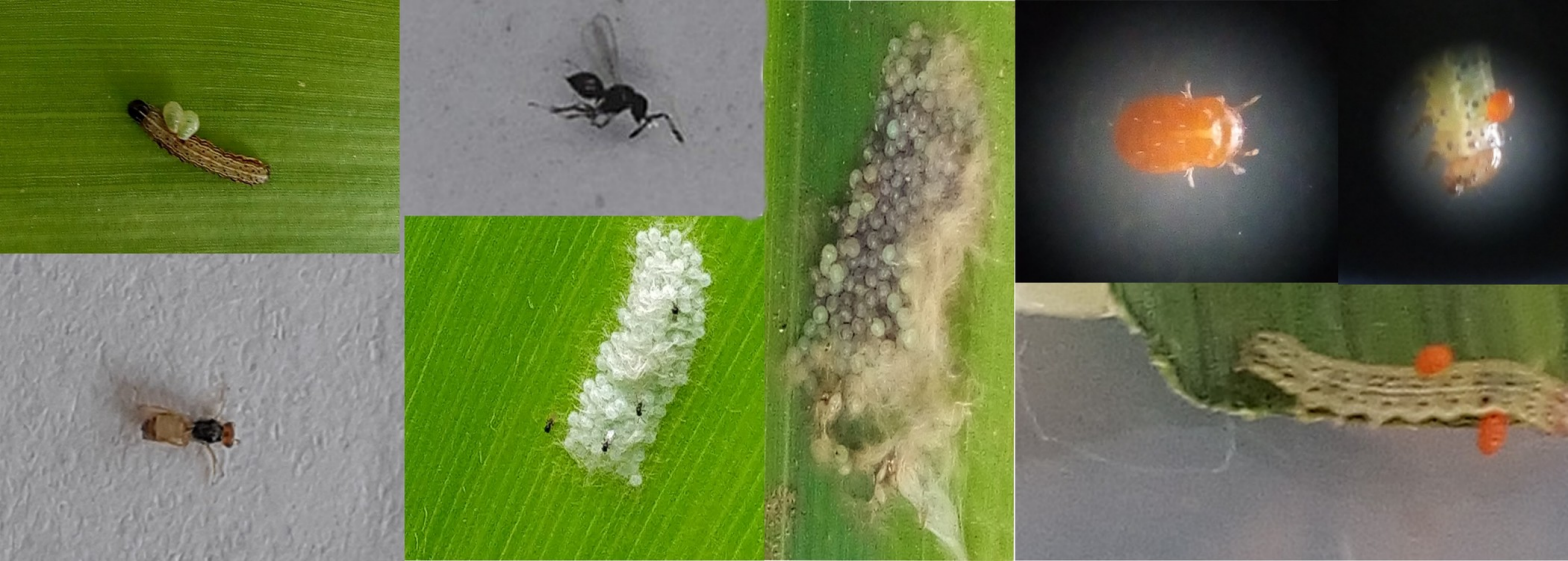
Natural enemies of Fall Armyworm (FAW) observed on FAW eggs and larvae. (a) *Euplectrus laphygmae* Adult and larva (Green colour) on FAW and (b) *Telenomus remus* Adults on FAW eggs and blackened parasitized FAW eggs and (c) *Trombidium* sp. (orange colour) on FAW larva.

The FAW individuals were also inspected for apparent signs of natural enemy attacks (Fig 1) and subsequently collected in separate containers.

The sampled egg masses were reared directly on the leaves on which they were laid in 170 mL plastic containers covered with meshed (< 0.1mm) lid at the Entomology laboratory of the Nigeria Agricultural Quarantine Service. The rearing conditions consisted of a mean temperature and relative humidity of 30±2°C and 82±2%, respectively under 12 hr ambient light conditions. The collected larvae were also reared individually in 250 mL glass containers with meshed lid and fed daily with 5–10 pieces of 2–3 cm maize leaves (depending on the life stage) until pupation. The eggs and larvae were observed daily for mortality and the emergence of natural enemies. The emerged FAW adults were visually identified and fed daily with 10% glucose solution in cotton wool and used for mass production of eggs and larvae. The egg parasitoids that emerges from FAW were fed with small droplets of honey placed at the side of the container while larval parasitoids were fed with a combination of 10% (w/v) glucose and 0.01% (w/v) ascorbic solution [2] in cotton wool. Some of the emerged egg and larval parasitoids were sampled and preserved in 70% (v/v) ethanol at room temperature for identification purpose.

### 2.2 Efficacy of parasitoids and predators on FAW

The parasitoids that emerged from FAW eggs and larvae were sampled, then introduced to the corresponding non-infested FAW eggs and larvae of FAW 24 hr after emergence. Ten unsexed adults of egg-parasitoid were exposed for 24 hr to 1 to 2 days old eggs of FAW obtained from laboratory reared adults. The FAW eggs (100) in four replicates were mounted on a 2 x 2 cm white cardboard painted with 20% (v/v) gum arabic (20:80 gum:water) to prevent them from falling-off. This process was repeated daily for 5 days with newly emerged parasitoids and 24–48 hr FAW eggs. After exposure, cards containing the eggs were placed in 170 ml containers under laboratory conditions until adult parasitoid or FAW larvae emerge as observed with a 10x magnifying lens. Developmental time from egg to adult parasitoid, number of dark (black) FAW eggs and emerged individuals relative to the number of eggs mounted on the cardboard was used to estimate the level of parasitism. Numbers of dark (i.e. parasitized) FAW eggs were counted by placing the card from the 4^th^ day after exposure under a stereomicroscope. The numbers of either emerged FAW larvae or adults parasitoids weredetermined by first killing them in the freezer (0–2°C).

Ten unsexed adult larval-parasitoids were introduced to ten (10) 1^st^-2^nd^ instar, 3–4 days old FAW larvae. After 24 hr post-introduction, the FAW larvae were removed into another 250 ml container. This was repeated with another set of 10 FAW larvae and an equal number of parasitoids for 48 hr. The parasitized FAW larvae were kept singly in containers and fed with fresh maize leaves daily until their mortality. The dead parasitized FAW larvae were kept in these same containers under laboratory conditions until adult parasitoid emergence. The parasitoids were also exposed to the different instars of FAW larvae as described above but because of cannibalistic behaviour of the pest, the 3^rd^ - 6^th^ FAW instars were placed singly in containers. Developmental time of the parasitoids from eggs to adults, number of parasitoids larvae on FAW larvae (used to estimate number of eggs laid by the parasitoid), and time of death of parasitized FAW larvae were recorded. The eggs and larvae from the same batches as the parasitized life stages that were not introduced to parasitoids were used as controls. Mites were introduced to 1^st^ -2^nd^ FAW larvae instars to determine the rate of parasitism or predation.

### 2.3 Identification of FAW natural enemies

#### 2.3.1 Morphological identification of parasitoids and mite

Morphological identification of the parasitoids and parasitic mite was conducted by Dr. G. Goergen at the Biodiversity Resource Center, International Institute of Tropical Agriculture (IITA), Cotonou, Benin. Voucher specimens of egg- parasitoids, larval-parasitoids, and parasitic mites were archived in the Nigeria Agricultural Quarantine Service and Biodiversity Resource Center, International Institute of Tropical Agriculture (IITA), Cotonou, Benin.

#### 2.3.2. Sequence and phylogenetic analyses of parasitoids

Due to inadequate mite samples and the need to further assess their role as natural enemies of FAW, only the parasitoids were sent for molecular analysis. Molecular identifications were carried out at CABI, UK on the parasitoids using cytochrome c oxidase subunit I (COI) DNA barcoding. A proprietary formulation [microLYSIS®- PLUS (MLP), Microzone, UK] was subjected to the rapid heating and cooling of a thermal cycler (An initial step of 65°C for 45 minutes, was followed by heating further to 96°C / 2 min; cooling to 65°C / 4 minutes; 96°C / 1 min; 65°C / 2 min; 96°C / 30 s and a final holding step at 20°C), to lyse cells and release ‘total’ cellular deoxyribonucleic acid (DNA). Following DNA release, Polymerase Chain Reaction (PCR) was employed to amplify copies of the COI barcode from the mitochondrial DNA (mtDNA) using standard primers LCO1490 and HCO2198 (5′-GGTCAACAAATCATAAAGATATTGG-3′ and 5′-TAAACTTCAGGGTGACCAAAAAATCA-3′, respectively; [14]. Amplifications were undertaken in 0.5 ml microcentrifuge tubes in 20 μl reactions containing: 1 μl MLP DNA extract; primers each at 150 nM; and 10 μl of mastermix solution (MegaMix-Royal [Microzone Ltd, UK], containing optimised mixture of *Taq* polymerase in 2 × Buffer (6 mM MgCl_2_), with 400 μM dNTPs and blue MiZN loading dye. Reactions were made up to a final volume of 20 μl sterile molecular grade H_2_O. PCR reactions were preincubated for 5 min at 95°C followed by 39 cycles of: 30 s at 94°C; 30 s at 51°C; 75 s at 72°C. This was followed immediately by a final extension step of 10 min at 72°C and then cooled to 10°C. The quality of the PCR product was assessed by undertaking gel electrophoresis. PCR purification step was carried out using microCLEAN purification solution (Microzone Ltd., UK) in accordance with the manufacturer’s instructions to remove unutilised dNTPs, primers, polymerase and other PCR mixture compounds and obtain a highly purified DNA template for sequencing. Purified PCR products were resuspended in 15 μl sterile molecular grade H_2_O. This procedure also allows concentration of low yield amplicons. Sequencing reactions were undertaken using BigDye® Terminator v3.1 kit from Applied Biosystems (Life Technologies, UK) and contained the following, in 0.5 ml microcentrifuge tubes: 2.68 μl of template DNA prepared as above; Primer HCO2198 at 320 nM; 5x BigDye® Terminator Sequencing Buffer; BigDye® Terminator. The sequencing reactions were preincubated for 1 min at 96°C followed by 25 cycles of: 20s at 96°C; 10s at 50°C; 4 min at 60°C. Samples were finally chilled at 10°C. Removal of excess unincorporated dye terminators was carried out using DyeEx™ 2.0 (Qiagen, UK). Dye removal was followed by suspension of the purified products in 16ml of highly deionised formamide Hi-Di™ (Life Technologies, UK) to prevent rapid sample evaporation and secondary structure formation [15]. Samples were loaded onto an AB 3130 Genetic Analyzer (Life Technologies, UK) and sequencing undertaken. Sequence trace files were assessed for quality using Sequencing Analysis Software v5.4 (Life Technologies, UK) and exported as text files.

After sequencing, identifications were undertaken by comparing the reverse complement of the sequence obtained, with those available from the Barcode of Life Data System (BOLD) (http://www.boldsystems.org/index.php/databases). Sequences were submitted to NCBI GenBank (https://submit.ncbi.nlm.nih.gov/subs/genbank/) and numbers assigned.

### 2.4 Data analysis

Data collected were analysed using the general linear model procedure of Statistical Analysis System [16]. Means of treatments and replicates were compared using Student-Neuman-Keuls and Least Significant differences (LSD) at 5%.

## 3. Results

### 3.1. Parasitoids identification and generation

Based on morphological characteristics, *Euplectrus laphygmae* (Hymenoptera.: Eulophidae)—a larval parasitoid, *Telenomus remus*, Nixon (Hymenoptera: Platygastridae)—an egg parasitoid, and *Trombidium* sp. (Acari.: Trombidiidae)—a parasitic mite, were each identified from infested field-collected FAW life stages (Fig 1). *Cotesia* or *Apanteles* spp. were also inferred to occur since *Stictopisthus* sp. (Hym.: Ichneumonidae), a secondary parasitoid, that attacks cocoons of Microgasterinae (e.g. *Cotesia*, *Apanteles* etc.) emerged from cocoon of larvae collected from the field (data not included). Numerous species of yet to be identified predators such as ladybird beetles, ants, earwigs, praying mantis were also observed occupying whorl and leaves of FAW damaged and undamaged maize plants (data not included).

A complete generation of *T*. *remus* from egg to adult emergence took 9–10 days after introduction to FAW eggs as recorded for five generations under laboratory conditions. In the laboratory, 100% parasitism by the introduced 10 unsexed egg-parasitoid occurred on the 100 FAW eggs with the emergence of *Telenomus* adults irrespective of the duration of exposure and parasitoid generation used. FAW larvae did not emerge from any of the already parasitized eggs collected from the field or those newly exposed to the parasitoid in the laboratory.

A 7–8 days developmental cycle (egg to adult emergence) was recorded for *E*. *laphygmae* after the parasitoid was introduced to FAW larvae. *Euplectrus laphygmae* adults typically oviposit on FAW larvae cuticles, depositing its non-apparently visible eggs in the process (Goergen Georg. IITA, personal communication). Based on the number of gregarious *E*. *laphygmae* larvae that emerged from the parasitized FAW, it was found that the larger the FAW instars, the greater the number of *E*. *laphygmae* larvae. At 1 day post-introduction (1DPI), only the number of eggs laid by *E*. *laphygmae* per instar increased, though not significantly compared to 2 days post-introduction (Table 2). The mean number of *E*. *laphygmae* eggs ranged from 1.50 on 2^nd^ instar to 5.00 on 4^th^ instar FAW larvae which was significantly different (P≤0.05) at 1DPI. At 2DPI, number of eggs laid per instar was significantly higher at the 3^rd^ and 4^th^ instar than 1^st^, 2^nd^, 5^th^ and 6^th^ instar stages. At 1^st^, 5^th^ and 6^th^ instars stages, parasitism did not occur and was low at 1DPI and 2DPI respectively (Table 2). The hatched eggs of *E*. *laphygmae* developed into gregarious green-yellowish green parasitoids larvae between 1–2 days post-introduction, killing the FAW host within 3 days. Brown pupae with loosely woven silken cells (cocoons) were observed at the 4^th^ days post-introduction and these emerged into adults by 7–8 days post-introduction.

**Table 2:** Mean number of emerged larvae of *Euplectrus laphygmae* per fall armyworm (FAW) instar after 1- and 2- days post-introduction (DPI).

FAW instar	Number of parasitoid larvae		LSD
	1 DPI	2 DPI	
1	0.00d	0.25c*	0.61
2	1.50c	2.00b*	1.22
3	4.50b	4.75a*	0.93
4	5.00a	5.50a*	0.71
5	0.00d	0.25c*	0.61
6	0.00d	0.00c*	0.00

DPI: days post introduction

Means followed by the same letter in the column are not significantly different (P≤0.05) according to Student-Neuman-Keuls

Means along the rows are not significant (*) according to Fisher’s Least Significant differences (LSD) at (P≤0.05)

Individuals of *Trombidium* sp., a parasitic mite, were observed to infest 1-3^rd^ instar of FAW larvae in the field. In the laboratory, the mites were initially found to be ectoparasitic on their FAW host but they were later observed to drop off the host after 4–8 days and were mobile. The ectoparasitic stages reduced the feeding, development and movement of FAW larvae, resulting in increasing paleness of the FAW larvae host. The mobile stage of the mites appears to be predatory resulting in the death of FAW 1^st^ instars within 24 hours of introduction.

### 3.2 Sequence analysis

Sequences of the parasitoids submitted in NCBI GenBank were assigned accession Nos. MT949366 (*Telenomus remus*) and MT949366 (*Eulophidae*). After manually correcting the sequences obtained in the present study, each was compared with authenticated sequences obtained from the Barcoding of Life Datasystem (BOLD; http://www.boldsystems.org/ [17]). This database includes publicly available and ‘private’ sequences that are not available for download.

#### 3.2.1. Identification and phylogenetic analysis of DAS-006-20-1 (MT949366)

**Fig 2:**
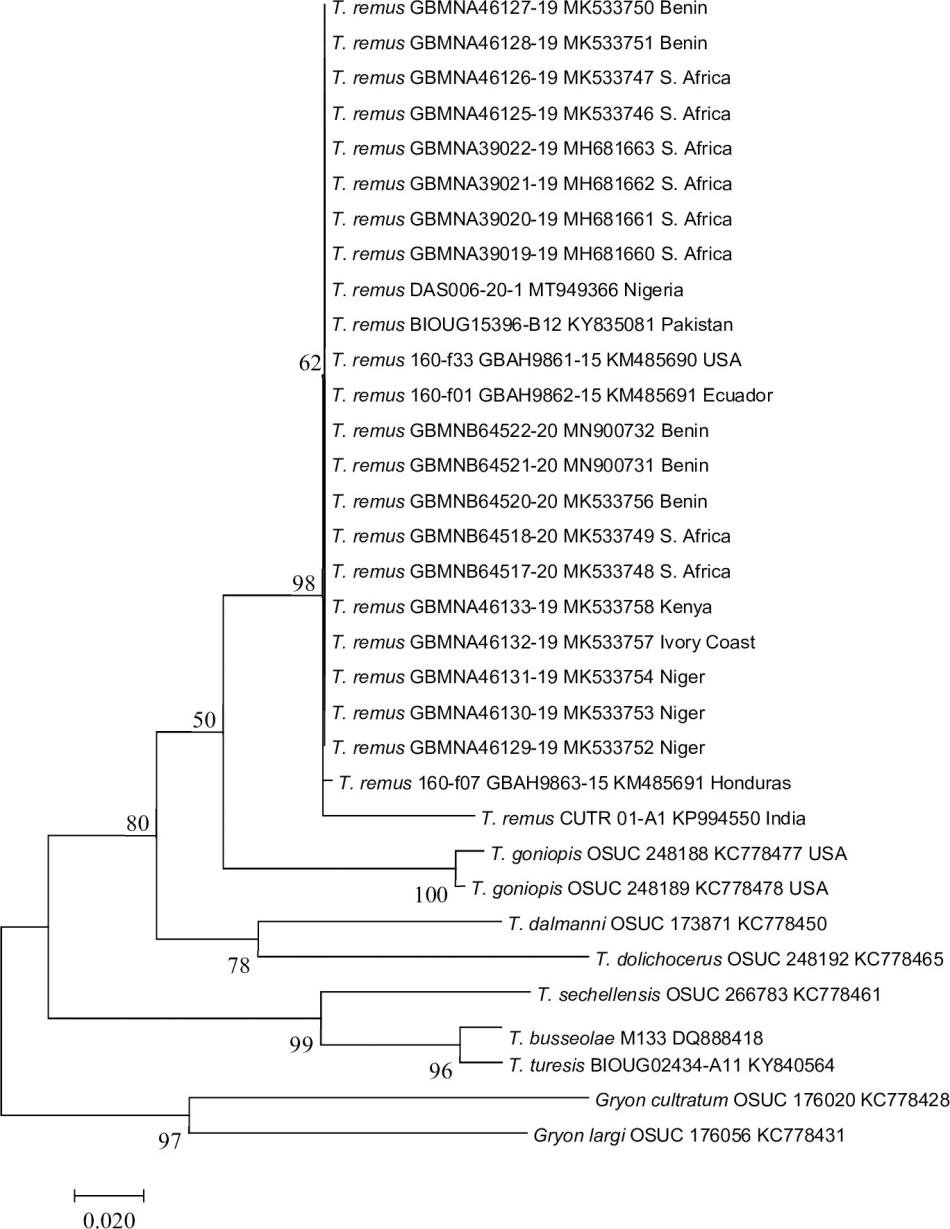
Molecular phylogenetic analysis of DAS-006-20-1 and related taxa by Maximum Likelihood method.

Following initial screening via BOLD which gave all matches >99% to be *Telenomus remus* (including top matches of 99.65% identity), phylogenetic analysis was undertaken to compare this sequence against selected voucher specimen-derived sequences of *T*. *remus* and related taxa and were downloaded from BOLD. The evolutionary history was inferred by using the Maximum Likelihood method based on the Tamura-Nei model [18]. The tree with the highest log likelihood (-1893.59) is shown (Fig 2). The percentage of trees in which the associated taxa clustered together is shown next to the branches. Initial tree(s) for the heuristic search were obtained automatically by applying Neighbor-Join and BioNJ algorithms to a matrix of pairwise distances estimated using the Maximum Composite Likelihood (MCL) approach, and then selecting the topology with superior log likelihood value. The tree is drawn to scale, with branch lengths measured in the number of substitutions per site. The analysis involved 33 nucleotide sequences. Codon positions included were 1st+2nd+3rd+Noncoding. All positions containing gaps and missing data were eliminated. There were a total of 379 positions in the final dataset. Evolutionary analyses were conducted in MEGA7 [19]. This showed that *T*. *remus* from Nigeria is closely related to those from Nigeria’s neighbour (Benin and Niger) as well as South Africa, amongst other locations (Fig 2).

#### 3.2.2 Identification of DAS-006-20-2 (MT949367)

Screening against the contents of BOLD did not result in a species-level identification for this specimen. Indeed, there was a single match at 99.33% to a sequence from a private sequence from the family Eulophidae. There were no other matches >95%. All matches >93% were to other sequences from the Eulophidae. As all the top matches were ‘private’ and are not present in GenBank, it is not possible to undertake a sensible phylogenetic analysis. Instead, the results of the BOLD identification are shown below (Table 3). However, the larva parasitoids are most likely to belong to the family Eulophidae as recorded by the high similarity with the private sequence from BOLD.

**Table 3:** BOLD identification of DAS-006-20-2 (MT949367) showing family and percent similarity to private sequences in the BOLD database.

Phylum	Class	Order	Family	Genus	Species	Subspecies	Similarity (%)	Status
**Arthropoda**	**Insecta**	**Hymenoptera**	**Eulophidae**				**99.33**	**Private**
**Arthropoda**	**Insecta**	**Hymenoptera**	**Eulophidae**				**94.3**	**Private**
**Arthropoda**	**Insecta**	**Hymenoptera**	**Eulophidae**				**94.13**	**Private**
**Arthropoda**	**Insecta**	**Hymenoptera**					**94**	**Private**
**Arthropoda**	**Insecta**	**Hymenoptera**	**Eulophidae**				**93.97**	**Private**
**Arthropoda**	**Insecta**	**Hymenoptera**	**Eulophidae**				**93.8**	**Private**
**Arthropoda**	**Insecta**	**Hymenoptera**	**Eulophidae**				**93.74**	**Private**
**Arthropoda**	**Insecta**	**Hymenoptera**	**Eulophidae**				**93.17**	**Private**
**Arthropoda**	**Insecta**	**Hymenoptera**	**Eulophidae**				**93**	**Private**
**Arthropoda**	**Insecta**	**Hymenoptera**	**Eulophidae**				**92.96**	**Private**
**Arthropoda**	**Insecta**	**Hymenoptera**	**Eulophidae**				**92.83**	**Private**
**Arthropoda**	**Insecta**	**Hymenoptera**	**Eulophidae**				**92.8**	**Private**
**Arthropoda**	**Insecta**	**Hymenoptera**	**Eulophidae**				**92.8**	**Private**
**Arthropoda**	**Insecta**	**Hymenoptera**	**Eulophidae**				**92.8**	**Private**
**Arthropoda**	**Insecta**	**Hymenoptera**	**Eulophidae**				**92.8**	**Private**
**Arthropoda**	**Insecta**	**Hymenoptera**	**Eulophidae**				**92.8**	**Private**
**Arthropoda**	**Insecta**	**Hymenoptera**	**Eulophidae**				**92.8**	**Private**
**Arthropoda**	**Insecta**	**Hymenoptera**	**Eulophidae**				**92.8**	**Private**
**Arthropoda**	**Insecta**	**Hymenoptera**	**Eulophidae**				**92.8**	**Private**
**Arthropoda**	**Insecta**	**Hymenoptera**	**Eulophidae**				**92.8**	**Private**

## 4. Discussion

Responses to the invasion of FAW in Nigeria and other countries in Africa have been focussed largely on advising farmers to deploy synthetic insecticides [13, 20–23] hitherto not used in maize cultivation. Unfortunately, synthetic insecticides are cost-prohibitive for the largely subsistent farmers, hence most have adopted different local practices to curtail the pest. Some farmers have also observed anecdotally that stoppage of insecticide application may promote the build-up of natural enemies to take over the management of FAW [20–24]. Since FAW is new to Nigeria and other African countries, identifying parasitoids and predators of *S*. *frugiperda* occurring in Nigeria, and documenting their life cycle and direct impacts will provide bases for actions required to ensure effective, sustainable, safe and easily adoptable FAW integrated pest management.

Of the over 150 parasitoids and predators reportedly used for biological control of FAW in its native tropical and subtropical regions of the Americas [5, 8], this study was able to provide evidence for the first time of the occurrence of two parasitoids and a parasitic/predatory mite in Nigeria. *Telenomus remus*, an egg parasitoid; *Euplectrus laphygmae*, a larval parasitoid and *Trombidium* sp. (tentative identification), a parasitic/predatory mite were observed suppressing the population of *Spodoptera frugiperda* in the field, as similarly reported in other African countries [2, 3, 11–13].

Kenis *et al*. [13] recommended that the present distribution of *T*. *remus* on the Africa continent should be assessed and the parasitoid could be introduced/imported into countries where it is absent. The results of this study showed the natural occurrence of *T*. *remus* in Nigerian maize fields and a demonstration of their parasitism efficacy, thus rendering a new introduction of the egg-parasitoid into the country unnecessary. The efficacy of *T*. *remus* was previously demonstrated by Queiroz *et al*. [25] with 5 females to 100±20 FAW eggs with nearly 100% parasitism irrespective of age of the parasitoid and FAW eggs, and duration of exposure. A similar conclusion was reached from the present study despite using 10 unsexed egg-parasitoids but similar number of FAW eggs: that *T*. *remus* can maintain their parasitism and efficiency irrespective of their age and number. This phenomenon is due to the ability of *T*. *remus* to adjust egg production based on host availability [25].

Despite knowledge of, and access to, information from the organization regulating the importation of bio-control agents for Nigeria, there is no record of *Telenomus* sp. introduction through importation. Thus, we can only hypothesize that it might have arrived in Nigeria either through neighboring countries from the sequence analysis in this study and as recorded by [13] or that it might have been present in the country prior to the FAW introduction. The fact that *T*. *remus* also attacks other *Spodoptera* species present in Nigeria support the latter hypothesis. The findings from this study therefore agrees with the suggestion of Kenis *et al*. [13] on investigating the host range of *Telenomus* spp. because this will assist in the understanding of potential deployment strategies for the parasitoid against FAW.

The larval parasitoid observed in this study will be referred to as *Euplectrus laphygmae* (Eulophidae) based on the morphological identification, since the information provided by the molecular analysis could only identify it only to the family level mainly due to inaccessibility of the private sequence in the GenBank. This also demonstrates the need for further taxonomic research and DNA barcoding of authenticated material to increase the coverage of definitive barcodes for arthropod species in the global databases. *Euplectrus laphygmae*, another FAW parasitoid detected in this study, has been recorded previously as existing naturally in Nigeria, Benin, Kenya, Congo, Cameroon, Cote d’voire, Uganda, Senegal, Sudan, Malawi, Zimbabwe and Tanzania, [26–29]. The genus *Euplectrus* are found all over the world with 209 species described so far [26, 30]. The larvae of *Euplectrus* species develop as gregarious ectoparasitoids on the lepidopterans Arctiidae, Erebidae, Geometriidae, Hesperiidae, Lasiocampidae, Limacodidae, Noctuidae, Papilionidae, Pyralidae and Tortricidae [28, 29]. *Euplectrus laphygmae* is also associated with several noctuid moths e.g. *Spodoptera littoralis* [31], *S*. *exigua*, *S*. *exempta*, *Helicoverpa* sp *and Chrysodeixis acuta* [27, 29, 31, 32], all common insect pests of vegetable crops in Nigeria. However, *E*. *laphygmae* had not yet been described in association with larvae of *S*. *frugiperda* globally. Previous studies reported *E*. *plathypenae* [33] and *E*. *ronnai* [34] *on* maize and *E*. *furnius* on rice [35] and maize [8], causing mortality of *S*. *frugiperda* larvae. This is the first report of *E*. *laphygmae* parasitizing *S*. *frugiperda* larvae on maize in Nigeria.

In addition to the FAW parasitoids, mites of the family Trombidiidae and Erythraeidae (Acari: Prostigmata) are ectoparasitic in their larval stage and predatory in their deutonymphal and adult stages on insects [36–38]. There are no records of larvae of Trombidiidae parasitising thrips, although several species of Trombidium Fabricius were collected on Coleoptera, Orthoptera, Diptera, Lepidoptera, Hemiptera, Homoptera, Hymenoptera, Opilionides, Pseudoscorpionides and Araneae [37, 39] such as Beetles, Aphids, Housefly, Grasshopper, Cricket etc. [36, 38] and arachnids [38]. This mite, *Trombidium* sp. (Acari.: Trombidiidae), to the best of our knowledge has never been recorded on *S*. *frugiperda* anywhere in the world until now. Molecular characterization will commence as soon as we fully understand its biology and mode of parasitism which will require us to obtain additional samples.

The development cycle of *Telenomus remus*. and *Euplectrus laphygmae* resulted in mortality of the FAW eggs and larvae respectively within the 1^st^ three days. In contrast, the mites mainly caused paleness, reduced feeding and growth (parasitic stage) and death (predatory stage) of the FAW larvae as previously documented [36, 37]. Even though the data presented herein were obtained under laboratory conditions, our results indicate the potential importance of these natural control agents in reducing the population density of *S*. *frugiperda*, which could be exploited in the field under appropriate conditions.

Appropriate conducive conditions provided in the field such as farm practices, biodiversity of the natural flora, shelter, food source, no or limited use of synthetic pesticides, etc. are important for the survival of diverse natural enemies and their attraction to and colonization of the host habitat location [6, 40, 41]. The above-mentioned conditions might constitute a “Push-Pull” strategy that led to the ecological niche occupation of the natural enemies found in this study. Also, the majority of the parasitoids were collected in maize plants with signs of FAW damage relative to the undamaged and slightly damaged plants. Interestingly, the heavily damaged plants were often occupied by larvae in the 5-6^th^ instar, which are stages not preferred by the parasitoid. Herbivore-induced plant volatiles emitted by caterpillar-damaged maize have been suggested to function as indirect defence signals that attract natural enemies to the microhabitat of herbivores [42–44]. This odour (volatiles) production could be further exploited in future studies as lures in attracting parasitoids especially using suitable maize varieties, frass and other wastes resulting from FAW consumption of the maize plant in providing simple adoptable techniques for farmers in order to discourage the use of synthetic insecticides.

In conclusion, this study has confirmed the existence in Nigeria of two parasitoids (*Euplectrus laphygmae* and *Telenomus remus*) and a parasitic/predatory mite (*Trombidium* sp.) attacking FAW which makes them candidates for inclusion in IPM tactics for the sustainable management of FAW. Field trials to validate the laboratory results along with mass production and release of these biological control agents should be considered to keep the FAW pest below economically damaging population levels. The use of these natural enemies would also help limit the use of synthetic insecticides that are hazardous to human health and the environment.

## References

[1] IAR&T. Outbreak of Armyworm, Spodoptera frugiderda (Smith) on maize fields in Nigeria. Report of survey and immediate control measures: The case of six states in Nigeria. Institute of Agricultural Research and Training, Obafemi Awolowo University, Moor Plantation, Ibadan, Nigeria. July 2016. ISBN 978-978-48780-8-1

[2] Prasanna BM, Huesing JE, Eddy R, Peschke VM (eds). Fall Armyworm in Africa: A Guide for Integrated Pest Management. First Edition. Mexico, CDMX: CIMMYT.2018.109p.

[3] Sisay B, Simiyu J, Mendesil E, Likhayo P, Ayalew G, Mohamed S. Fall armyworm, Spodoptera frugiperda infestations in East Africa: assessment of damage and parasitism. Insects 2019; 10: 195. doi: 10.3390/insects10070195 31277232 PMC6681394

[4] Tefera T, Goftishu M, Ba M, Muniappan R. A Guide to Biological Control of Fall Armyworm in Africa Using Egg Parasitoids. First Edition, Nairobi, Kenya. 2019.

[5] Molina-Ochoa J, Carpenter JE, Heinrichs EA, Foster JE. Parasitoids and parasites of Spodoptera frugiperda (Lepidoptera: Noctuidae) in the Americas and Caribbean Basin: An inventory. Fla. Entomol. 2003; 86: 254–289.

[6] Murua MG, Molina-Ochoa J, Fidalgo P. Natural distribution of parasitoids of larvae of the fall armyworm, *Spodoptera frugiperda*, in Argentina. Journal of Insect Science 2009; 9:20–37. https://academic.oup.com/jinsectscience/article-abstract/9/1/20/891778. doi: 10.1673/031.009.2001 19613463 PMC3011829

[7] Estrada VO, Cambero Campos J, Robles Bermudez A, Rios Velasco C, Carvajal Cazola C, Isiordia Aquino N, et al. Parasitoids and entomopathogens of the fall armyworm Spodoptera frugiperda (Lepidoptera: Noctuidae) in Nayarit, Mexico. Southwestern Entomol. 2013; 38: 339–344.

[8] Sturza VS, Dequech STB, Tavares MT, Guths C, Walker MP, Bolzan A. Euplectrus furnius parasitizing Spodoptera frugiperda in maize in Brazil. Ciência Rural, Santa Maria 2013; 43(11): 1958–1960.

[9] Meagher RL, Nuessly GS, Nagoshi RN, Hay-Roe MN. Parasitoids attacking fall armyworm (Lepidoptera: Noctuidae) in sweet corn habitats. Biol. Control 2016; 95: 66–72.

[10] Kenis M, Hurley B, Hajek AE, Cock M. Classical biological control of insect pests of trees—Facts and figures. Biol. Invasions 2017; 19: 3401–3417.

[11] Sisay B, Simiyu J, Malusi P, Likhayo P, Mendesil E, Elibariki N, et al. First report of the fall armyworm, Spodoptera frugiperda (Lepidoptera: Noctuidae), natural enemies from Africa. J. Appl Entomol. 2018; 142(8): 800–804. doi: 10.1111/jen.12534

[12] Agboyi LK, Mensah SA, Clottey VA, Beseh P, Glikpo R, Rwomushana I, et al. Evidence of Leaf Consumption Rate Decrease in Fall Armyworm, Spodoptera frugiperda, Larvae Parasitized by Coccygidium luteum. Insects 2019; 10: 410. doi: 10.3390/insects10110410 31744045 PMC6920753

[13] Kenis M, du Plessis H, Van den Berg J, Ba MN, Goergen G, Kwadjo KE, et al. Telenomus remus, a candidate parasitoid for the biological control of Spodoptera frugiperda in Africa, is already present on the continent. Insects 2019; 10:92. doi: 10.3390/insects10040092 30934941 PMC6523282

[14] Folmer O, Black M, Hoeh W, Lutz R, Vrijenhoek R. DNA primers for amplification of mitochondrial cytochrome c oxidase subunit I from diverse metazoan invertebrates. Mol. Mar. Biol. Biotechnol. 1994; 3(5):294–299 7881515

[15] Sambrook J, Friitsch EF, Maniatis T. Molecular Cloning: A Laboratory Manual, vol. I. 2nd edition. Cold Spring Harbor Laboratory Press, USA. 1989.

[16] Statistical Analysis System (SAS). SAS user’s guide, 2009. SAS Institute.

[17] Ratnasingham S, Hebert PDN. BOLD: The Barcode of Life Data System (www.barcodinglife.org). Mol. Ecol. Notes 2007; 7:355–364. doi: 10.1111/j.1471-8286.2007.01678.x 18784790 PMC1890991

[18] Tamura K, Nei M. Estimation of the number of nucleotide substitutions in the control region of mitochondrial DNA in humans and chimpanzees. Mol. Biol. Evol. 1993.10:512–526. doi: 10.1093/oxfordjournals.molbev.a040023 8336541

[19] Kumar S, Stecher G, Tamura K. MEGA7: Molecular Evolutionary Genetics Analysis version 7.0 for bigger datasets. Mol. Biol. Evol. 2016; 33:1870–1874. doi: 10.1093/molbev/msw054 27004904 PMC8210823

[20] Kansiime MK, Mugambi I, Rwomushana I, Nunda W, Lamontagne-Godwin J, Rware H, et al. Farmer perception of fall armyworm (Spodoptera frugiderda J.E. Smith) and farm-level management practices in Zambia. Pest Manag. Sci. 2019; 75: 2840–2850. 10.1002/ps.5504 31148397 PMC6771660

[21] Kumela T, Simiyu J, Sisay B, Likhayo P, Mendesil E, Gohole L. et al. Farmers’ knowledge, perceptions, and management practices of the new invasive pest, fall armyworm (Spodoptera frugiperda) in Ethiopia and Kenya. Int. J. Pest Manag. 2018. 10.1080/09670874.2017.1423129.

[22] Tambo JA, Day RK, Lamontagne-Godwin J, Silvestri S, Beseh PK, Oppong-Mensah B. et al. Tackling fall armyworm (Spodoptera frugiperda) outbreak in Africa: an analysis of farmers’ control actions. International Journal of Pest Management. 2019. doi: 10.1080/09670874.2019.1646942

[23] Togola A, Meseka S, Menkir A, Badu-Apraku B, Bouka O, Tamò M. et al. Measurement of Pesticide Residues from Chemical Control of the Invasive Spodoptera frugiperda (Lepidoptera: Noctuidae) in a Maize Experimental Field in Mokwa, Nigeria. Int. J. Environ. Res. Public Health 2018; 15: 849–860. 10.3390/ijerph15050849 PMC5981888 29693596

[24] Bateman ML, Day RK, Luke B, Edgington S, Kuhlmann U, Cock MJW. Assessment of potential biopesticide options for managing fall armyworm (Spodoptera frugiperda) in Africa. J. Appl. Entomol. 2018; 142: 805–819. 10.1111/jen.12565

[25] Queiroz AP, Favetti BM, Luski PGG, Goncalves J, Neves MOJ, Bueno AF. Telenomus remus (Hymenoptera: Platygastridae) Spodoptera frugiperda (Lepidoptera: Noctuidae) eggs:different parasitoid and host egg ages.Semina:Ciencias Agrarias, Londrina 2019; 40 (6):2933–2946. Doi: 10.5433/1679-0359.2019v40n6Supl2p2933

[26] Ferrière C. New species of Euplectrini (Hym. Chalcidoidea) from Europe, Africa and Asia. Bulletin of Entomological Research 1941; 32(1): 17–48.

[27] Delvare G, Rasplus JY. Spodophagus, a new genus of Pteromalidae (Hymenoptera), for an important parasite of Spodoptera litoralis (Lepidoptera: Noctuidae) in Madagascar. Bulletin of Entomological Research 1994; 84: 191–197. 10.1017/S0007485300039687

[28] Zhu CD, Huang DW. A study of the genus Euplectrus Westwood (Hymenoptera: Eulophidae) in China. Zoological Studies 2003; 42(1): 140–164.

[29] Yefremova ZA. New records of the genus Euplectrus Westwood (Hymenoptera: Eulophidae) from Southeast Asia, South Asia and Oceania, with description of three new species and a key. Israel Journal of Entomology 2017; 47:55–85.

[30] Noyes JS. Universal Chalcidoidea Database (UCD). World Wide Web electronic publication. http://www.nhm.ac.uk/chalcidoids (Accessed Nov. 2019).

[31] Gerling D, Limon S. A biological review of the genus Euplectrus (Hym.: Eulophidae) with special emphasis on E. laphygmae as a parasite on Spodoptera littoralis (Lep.: Noctuidae). Entomophaga 1976; 21(2): 179–187. 10.1007/BF02371904.

[32] Fry JM. 1989. Natural enemy databank, 1987. A catalogue of natural enemies of arthropods derived from records in the CIBC Natural Enemy Databank. CAB International, Wallingford, Oxon, UK.

[33] Costa Lima CA. Insetos do Brasil XII. Himenópteros, 2ª parte (Serie Didática No. 14). Rio de Janeiro: Universidade Rural, Escola Nacional de Agricultura. 1962; 393p. Available from: <https://www.ufrrj.br/institutos/ib/ento/tomo12.pdf>. Accessed: 10th November, 2019.

[34] De Santis L. Nueva sinonimia, nueva combinación y nuevas citas de Himenopteros Calcidoideos para la República Argentina. Neotrópica 1980; 26(76):153–154.

[35] Pantoja A, Fux JR. Prevalence of biotic control agents in the fall armyworm Spodoptera frugiperda (J.E. Smith) (Lepidoptera: Noctuidae). Folia Entomológica Mexicana 1992; 84:79–84.

[36] Zhang Z-Q. Biology and ecology of trombidiid mites (Acari: Trombidioidea). Experimental and Applied Acarology 1998; 22:139–155.

[37] Goldarazena A, Zhang Z-Q, Jordana R. A new species and a new record of ectoparasitic mites from thrips in Turkey (Acari: Trombidiidae and Erythraeidae). Systematic Parasitology 2000; 45: 75–80. doi: 10.1023/a:1006289526619 10682925

[38] Gupta SK, Kumar PS. The underestimated worth of predatory and parasitic mites in India: does it really have to import exotic species for biological control? CAB Reviews 2018; 13(31). doi: 10.1079/PAVSNNR201813031

[39] Welbourn WC. Potential use of trombidioid and erythraeoid mites as biological control agents of insect pests. In Biological control of pests by mites, Hoy M.A., Cunningham G.L., and Knutson L. (eds), pp. 89–107. University of California Press/ANR Publishing Co, Oakland.1983.

[40] Kogan M, Gerling D, Maddox JV. Enhancement of Biological Control in annual agricultural environments. In: Bellows T. and Fisher T. (eds.). Handbook of Biological Control 1999. pp. 789–818. Academic Press.1999.

[41] Hoballah ME, Degen T, Bergvinson D, Savidan A, Tamo C, Turlings TCJ. Occurrence and direct control potential of parasitoids and predators of the fall armyworm (Lepidoptera.: Noctuidae) on maize in the subtropical lowlands of México. Agriculture and Forest Entomologist 2004; 6:83–88.

[42] Baehrecke EH, Vinson SB, Williams HJ. Foraging behaviour of *Campoletis sonorensis* in response to *Heliothis virescens* and cotton plants. Entomol. Exp. Appl. 1990; 55:57–58.

[43] Turlings TCJ, Tumlinson JH, Lewis WJ. Exploitation of herbivore-induced plant odours by host-seeking wasps. Science 1990; 250:1251–1253. doi: 10.1126/science.250.4985.1251 17829213

[44] McAuslane HJ, Vinson SB, Williams HJ. Stimuli influencing host microhabitat location in the parasitoid *Campoletis sonorensis*. Entomol. Exp. Appl. 1991; 58:267–277.

